# Natural killer cells from patients relapsing on daratumumab therapy express an exhausted-associated phenotype

**DOI:** 10.46989/001c.163405

**Published:** 2026-07-07

**Authors:** Katrine Fladeland Iversen, Line Nederby, Thomas Lund, Trine Andreasen Leth, Torben Plesner

**Affiliations:** 1 Institute of Regional Health Research, University of Southern Denmark; 2 Department of Internal Medicine, Section of Hematology, Lillebaelt Hospital, University Hospital of Southern Denmark, Beriderbakken 4, 7100 Vejle, Denmark; 3 Department of Biochemistry and Immunology, Lillebaelt Hospital, University Hospital of Southern Denmark, Beriderbakken 4, 7100 Vejle, Denmark; 4 Department of Hematology, Odense University Hospital, J.B. Winsløvs Vej 4, 5000 Odense C, Denmark

**Keywords:** Multiple myeloma, Daratumumab, NK cells, resistance, immunotherapy

## Abstract

The monoclonal antibody daratumumab can induce antibody-dependent cellular cytotoxicity (ADCC) via CD16 expressed by natural killer (NK) cells. Prior studies have shown that the number of NK cells decreased after initiation of daratumumab treatment. We used flow cytometry to evaluate the expression of immune checkpoint receptors on NK cells from patients with newly diagnosed multiple myeloma (NDMM) compared to myeloma patients progressing during treatment with daratumumab (DRMM). In accordance with prior studies, we found that the percentage of NK cells was significantly lower in the DRMM group compared to the NDMM group. In addition, the percentage of the cytotoxic CD56dim NK cell subset was lower in the DRMM group, and a lower portion of these NK cells expressed CD16, the receptor mediating ADCC. There was no difference in the expression of the inhibitory receptor PD-1, but the NK cells from DRMM patients expressed an exhausted-associated phenotype with a lower expression of the stimulatory receptor DNAM-1 and a higher expression of the inhibitory receptor TIGIT. Dysfunctional NK cells have been observed in other patients progressing on daratumumab. Our data show that patients progressing on DARA-containing regimens display NK cells with an inhibitory-skewed phenotype. Whether this reflects a driver, biomarker, or consequence of resistance requires further functional and longitudinal investigation.

## Introduction

Multiple myeloma (MM) is an incurable disease characterized by clonal accumulation of malignant plasma cells in the bone marrow (BM).^1^ Remarkable progress has been made in the treatment of MM with the introduction of numerous different immunotherapies.^2^ This has resulted in prolongation of overall survival.^3,4^ Despite this progress, MM remains an incurable disease.

**Figure 1.**
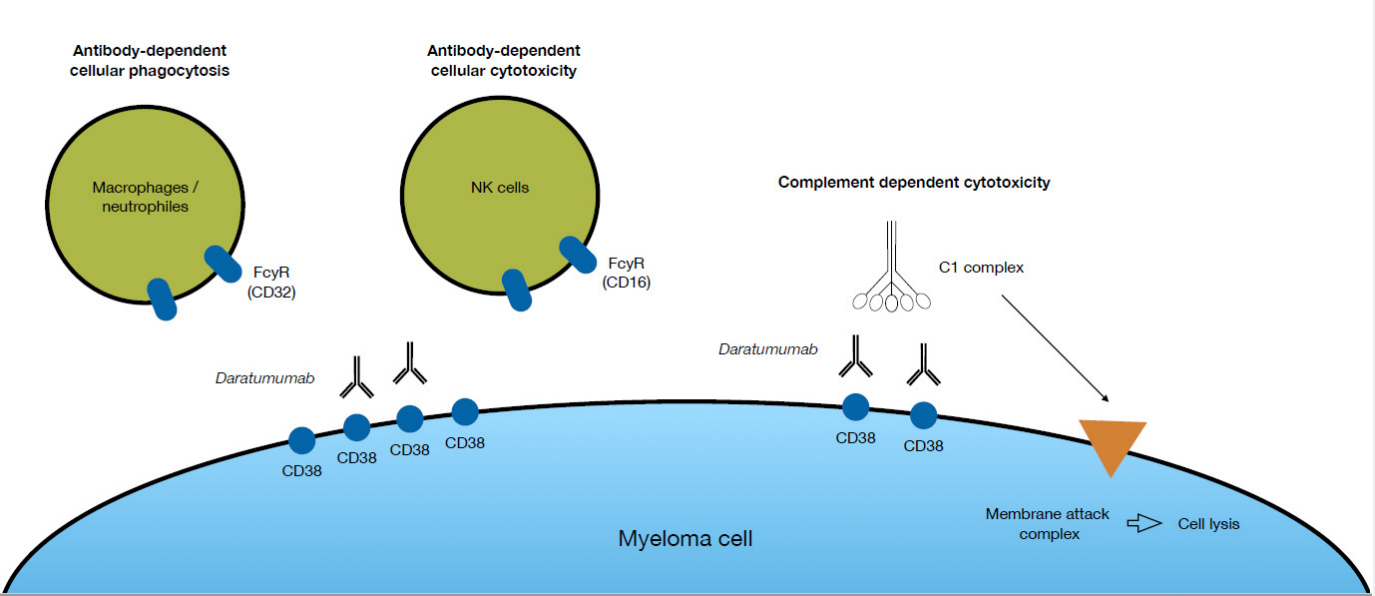
Daratumumab’s mode of action to directly kill tumor cells. Following the binding of daratumumab to CD38 on a myeloma cell, antibody-dependent cellular cytotoxicity is triggered by interactions between the Fc region of daratumumab bound to a myeloma cell and Fc receptors, particularly FcRI and FcRIII, on immune effector cells such as neutrophils, macrophages, and natural killer cells. Daratumumab-coated tumor cells are phagocytosed by macrophages or undergo cytolysis by NK cells. In the case of complement-dependent cytotoxicity, recruitment of C1q by daratumumab bound to the myeloma cell surface is an obligatory first step. This triggers a proteolytic cascade that leads to the formation of a membrane attack complex that kills the myeloma cell by cell lysis.

A significant improvement in the treatment of MM was the introduction of the first CD38-targeting monoclonal antibody daratumumab (DARA). The target of DARA, CD38, is highly expressed on malignant plasma cells and to a lesser extent on immune effector cells, including natural killer (NK) cells.^5^ DARA has three different modes of action to directly kill tumor cells (Fig. 1). Firstly, it can induce antibody-dependent cellular cytotoxicity (ADCC) via CD16 expressed by NK cells. Secondly, antibody-dependent cellular phagocytosis (ADCP), where the Fc region of DARA, bound to CD38 on MM cells, reacts with the FC receptor on effector cells, thus inducing phagocytosis of the MM cell.^6^ And, thirdly, complement-dependent cytotoxicity (CDC), where recruitment of the complement factor C1q by DARA bound to the myeloma cell surface triggers a proteolytic cascade that leads to the formation of a membrane attack complex that kills the myeloma cell by cell lysis.^7^ Beside these abilities to directly kill tumor cells, DARA affects the immune system through several different modes of action. Studies of patient material collected during clinical trials, where DARA was given as monotherapy, showed that DARA may eliminate CD38^+^ populations of regulatory T-cells, B-cells, and monocytes/macrophages that impose a break on the cytotoxic T-cells.^5^ Consequently, cytotoxic T-cells proliferate and become activated following treatment with DARA. DARA also inhibits MM cell adhesion to the bone marrow stromal cells, via CD38 internalization through the endocytic machinery rendering the MM cells more sensitive to concomitant therapy.^8^ Regardless of the multiple modes of action of DARA, the majority of patients receiving this antibody eventually relapse.^9^ The specific mechanism behind the development of resistance to DARA is not yet fully clarified, but NK cells could play a role.^10^

NK cells are a critical subset of the innate immune cells, as they act as first responders against viral infections, and play an important role in the immune surveillance of cancer. They can be classified into two major groups – CD56^bright^ and CD56^dim^. CD56^dim^ NK cells express CD16, which is the key mediator of ADCC.^11^ This makes this subset the one with most cytotoxic potential. Besides CD16, which is the most potent NK cell activating receptor, activation of NK cells also depends on the integration of other costimulatory and inhibitory signals from cell-surface receptors upon contact with ligands expressed on target cells. CD56^bright^ NK cells do not express CD16, hence they cannot initiate ADCC. They release pro-inflammatory cytokines and promote other components of the immune system through e.g. INFγ and TNFα production.^12^

Prior studies have shown that the number of NK cells (CD45^+^CD3^-^CD56^bright/dim^) decrease after initiation of DARA.^13,14^ This decrease is due to the fact that NK cells are, in part, CD38^+^ and, therefore, exposed to ADCC like MM cells.^13^ This decrease is more pronounced in the activated CD56^dim^ subset.^14,15^ Furthermore, it has been shown that expression of CD16 by the remaining NK cells is low.^16^ Information about the expression of immune checkpoint receptors on NK cells from MM patients progressing on DARA is very limited.

We studied the expression of inhibitory and costimulatory immune checkpoint receptors on NK cells isolated from peripheral blood (PB) of patients progressing on a DARA-containing regimen (Daratumumab Refractory Multiple Myeloma patients; DRMM). The results were compared with the profile of treatment-naive, newly diagnosed myeloma patients (NDMM).

## Materials and Methods

### Study Population and Sample Collection

Patients diagnosed with MM according to the International Myeloma Working Group (IMWG) guidelines at the Departments of Hematology at Vejle Hospital and Odense University Hospital were included in the study.^17^ They were either NDMM or DRMM patients.^18^ The study was approved by the regional Ethical Committee (S-20170212). Participation was voluntary, and written informed consent was obtained from all subjects. Samples were obtained between December 2019 and May 2021. Data on patient characteristics and prior treatment were retrospectively obtained from the electronic medical journal and, afterwards, registered in a designated Research Electronic Data Capture (REDCap) database.^19,20^

### Cell Isolation from PB samples

PB samples were obtained from NDMM (n = 13) and DRMM (n = 21). Ten milliliters of PB were collected in BD Vacutainer® EDTA blood collection tubes (BD Biosciences, San Jose, CA, USA). PB mononuclear cells (PBMCs) were isolated by density-gradient centrifugation using FicollPaque™ PLUS (GE Healthcare Bio-Sciences AB. Uppsala, Sweden) according to the manufacturer’s instructions. Cells were cryopreserved in a medium of 70% RPMI 1640 with GlutaMAX™ supplement (ThermoFisher Scientific, Waltham, MA, USA), 20% heat-inactivated fetal bovine serum (FBS) (ThermoFisher Scientific), and 10% dimethyl sulfoxide (Sigma-Aldrich, St. Louis, MO, USA), and kept at − 135°C until use. The isolation process was initiated less than 24 h after the collection.

### Flow Cytometry

Cryopreserved PBMCs were thawed in a 37°C water bath and resuspended in phosphate buffered saline (PBS) with 15% heat-inactivated FBS (ThermoFisher Scientific). Concentration and viability were determined using the trypan blue exclusion method and the Countess II Automated Cell Counter (ThermoFisher Scientific). The median viability in PBMC were 82% (range 69–98%). Cells in PBS/0.5% bovine serum albumin (BSA) were treated with Human TruStain FcX (BioLegend, San Diego, CA, USA) according to the manufacturer’s recommendations. Subsequently, monoclonal antibodies and cells were mixed in Brillant Stain Buffer Plus (BD Biosciences) and the suspensions were incubated for 15 min at room temperature. Antibodies (all from BD Biosciences) had been titrated using relevant materials: anti-CD45 BV650, anti-CD3 PerCP-Cy5.5, anti-CD16 R716, anti-CD56 PE, anti-CD19 PerCP-Cy5.5, anti-CD14 PerCP-Cy5.5, anti-CD15 PerCP-Cy5.5, anti-programmed cell death protein 1 (PD-1) PE-Cy7, anti-DNAX accessory molecule-1 (DNAM-1) BB515, and anti-T-cell immunoglobulin and ITIM domains (TIGIT) BV421 and 7-AAD. A fluorescence-minus-two (FM2) tube, leaving out anti-DNAM-1 BB515 and anti-PD-1 PE-Cy7, and a fluorescence-minus-one (FM1) tube, leaving out anti-TIGIT BV421, were prepared for each sample. After staining, samples were treated with 2 mL 1× BD Pharm Lyse™ Lysing buffer (BD Biosciences) and washed using PBS/0.5% BSA. Before analysis, 7-AAD (BD Biosciences) was added.

**Figure 2.**
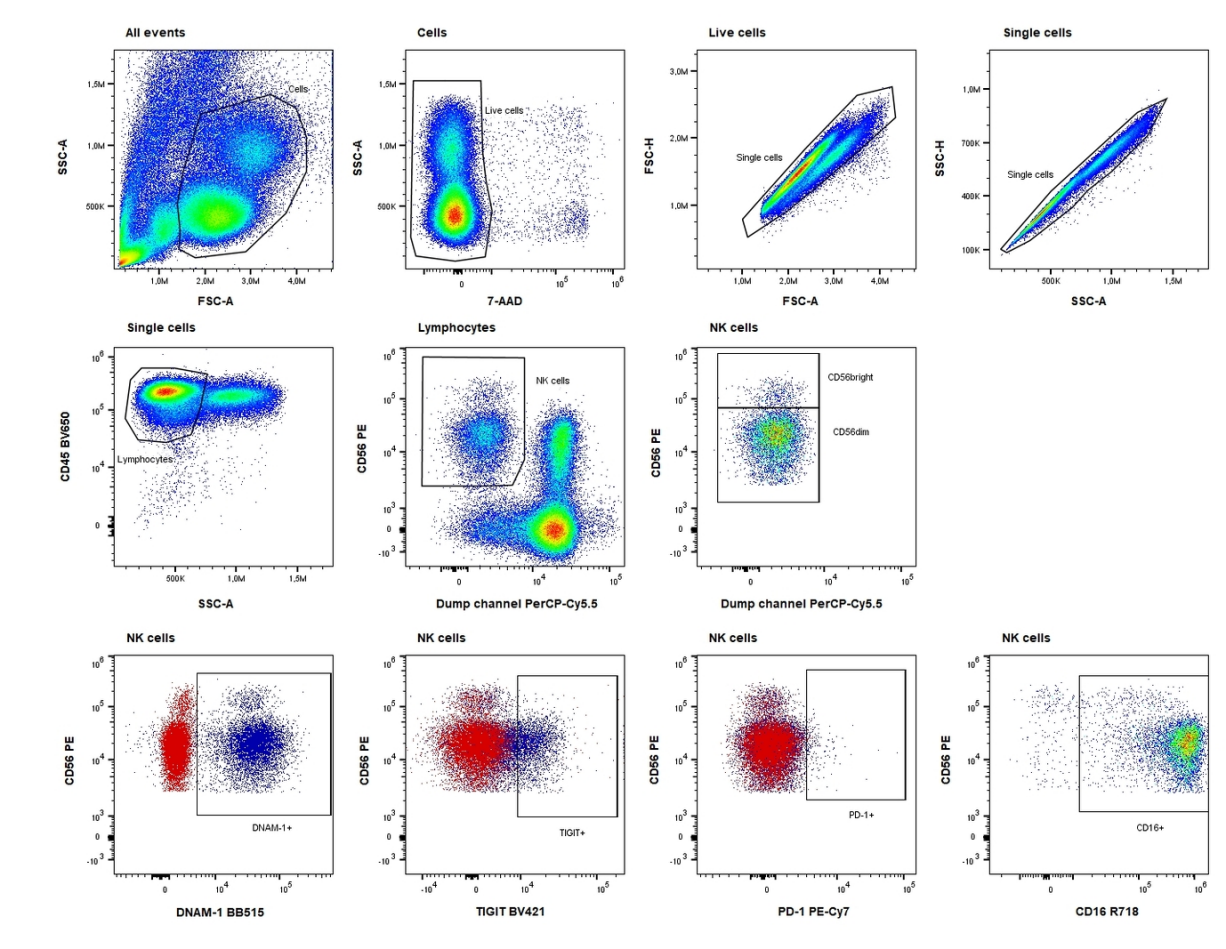
Representative patient sample illustrating the gating strategy used in the study. In the first panel, *all events* are shown in a Forward scatter (FSC)-area (A) / Side scatter (SSC)-area (A) plot, where the overall cell subset was defined. Next, live cells (7-AAD^-^) were selected for further analysis. In the following two panels, doublets were excluded using FSC-A / FSC-height (H) plot and SSC-A / SSC-H plot. As a next step, lymphocytes, defined as CD45^high^SSC^low^, were identified in a SSC-A / CD45 BV650 plot. Among the lymphocytes, NK cells (including the CD56^bright^ and CD56^dim^ subsets), were distinguished using CD56 PE in combination with a dump channel containing CD3, CD14, CD15 and CD19, all conjugated with PerCP-Cy5.5. The final panels show the expressing of DNAM-1, TIGIT, PD-1, and CD16 on the gated NK cells. Red subsets represent the control fluorescence minus 1- and 2 tubes, while blue subsets show the same sample, but stained with the full antibody panel.

Samples were analyzed immediately after staining on an ACEA NovoCyte Quanteon 4025 flow cytometer (Agilent Technologies, Inc. Santa Clara, CA, USA). Consistency and stability of the instrument were verified on a daily basis using NovoCyte 6 peak QC Particles (Agilent Technologies, Inc.). Compensation was performed using UltraComp eBeads™ (ThermoFisher Scientific) stained with the antibodies and cells stained with 7-AAD. Data were analyzed using FlowJo™ software version 10.7.2 (BD Biosciences). The FM2 and FM1 controls were used for objective gating of PD-1, DNAM-1, and TIGIT, and both the percentage of the positive subset and the median fluorescence intensity (MFI) of this positive subset were evaluated. The gating strategy is shown in Fig. 2.

### Statistical Analysis

Both the fraction of cells expressing a given marker and the MFI of each marker on NK cells were expressed as medians. Since the data sets were not normally distributed (tested by a q-q plot), a Mann Whitney U test was used for analysis of differences between groups. p values of less than 0.05 were considered significant. All statistical analyses were performed using STATA version 16.0 for PC (Stata Corp LP, College Station, TX, USA) and GraphPad Prism version 10.5.0 (GraphPad Software, Boston, MA, USA).

## Results

### Patient Characteristics

A total of 34 MM patients participated in the study: 13 NDMM and 21 DRMM. DRMM had received a median of four prior lines of therapy (Table 1).

**Table 1.** Patient characteristics

	*Patient group*	
	*NDMM* *n= 13*	*DRMM* *n= 21*
Age*; years; median (range)	80 (60-86)	61 (47-81)
**Gender**; n (%)		
Female	5 (38)	12 (57)
Male	8 (62)	9 (43)
Immunoglobulin subtype*; n (%)		
IgG	8 (62)	11 (52)
IgA	3 (23)	2 (10)
Light-chain only	2 (15)	8 (38)
Non secretory	0 (0)	0 (0)
ISS*; n (%)		
I	2 (15)	6 (29)
II	6 (46)	6 (29)
III	2 (15)	2 (10)
Unknown	3 (23)	7 (33)
ECOG performance status*; n (%)		
0	7 (54)	11 (52)
1	6 (46)	2 (10)
2	0 (0)	1 (5)
Unknown	0 (0)	7 (33)
**Fluorescence in situ hybridization**^§^; n (%)		
High-risk	2 (15)	5 (24)
Standard-risk	11 (85)	11 (52)
Unknown	0 (0)	5 (24)
**Number of prior lines of therapy**; median (range)	0 (0-0)	4 (0-15)
**Prior exposed to;** n (%)		
IMID	NA	15 (71)
PI	NA	21 (100)
Cyclophosphamid	NA	10 (48)
HDT + ASCT	NA	13 (62)
**Treatment;** n (%)		
Daratumumab + IMID	NA	7 (33)
Daratumumab + PI	NA	6 (29)
Daratumumab monotherapy	NA	3 (14)
Daratumumab + other	NA	5 (24)

*= at the time of diagnosis of multiple myeloma<br>

§= If assessed more than once, the most recent result prior to initiation of daratumumab treatment is shown. High-risk aberrations were defined by the presence of either t(4;14), t(14;16), or del17p, each detected with a cutoff of 10% according to international standards for cytogenetic evaluation. NDMM=newly diagnosed multiple myeloma patients; DRMM=daratumumab refractory multiple myeloma patients; ISS= international staging system; NA=not applicable; IMID= immunomodulatory imide drug; PI= proteasome inhibitor; HDT + ASCT = high dose treatment + autologous stem cell transplantation.

### Decreased percentage of NK cells in the DRMM group

We found that the percentage of NK cells out of the total number of lymphocytes was significantly lower in the DRMM group compared to the NDMM (median 5.2% versus 21.2%, *p*=0.002). In addition, the percentage of CD56^dim^ NK cells was significantly lower in the DRMM compared to the NDMM (median 85.5% versus 97.9%, *p*=0.0001).

**Figure 3.**
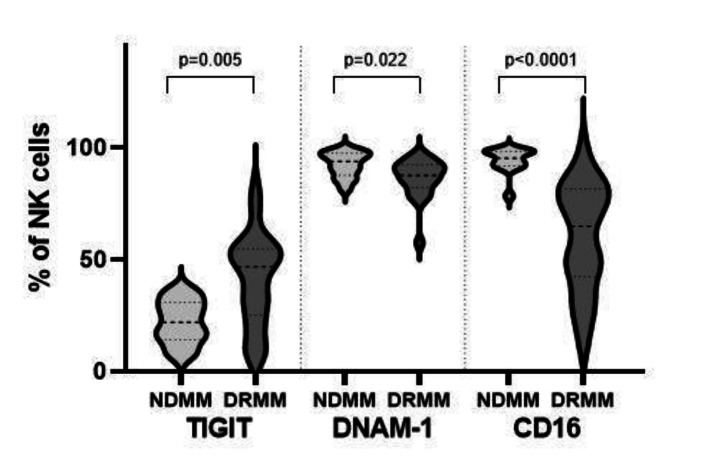
The expression of TIGIT, DNAM-1 and CD16 by NK cells from NDMM and DRMM. TIGIT, anti-T-cell immunoglobulin and ITIM domains; DNAM-1, anti-DNAX accessory molecule-1; NK cells, natural killer cells; NDMM, newly diagnosed multiple myeloma patients; DRMM, daratumumab-refractory multiple myeloma patients

The percentage of NK cells expressing CD16 was statistically lower in DRMM compared to NDMM (median 64.7% versus 95.1%, p=<0.0001) (Table 2 and Fig. 3). Moreover, the CD16 expression level of the NK cells was also lower in the DRMM group as determined by MFI (MFI 126,851 versus 466,000, p=0.0001). The same pattern was seen when analyzing CD56^dim^ subset: only 77.2% of the CD56^dim^ NK cells expressed CD16 in the DRMM group compared to 97.2% in the NDMM group (*p*=0.0001). Furthermore, a lower level of CD16 expression on the CD16^+^ CD56^dim^ NK cells was evident in the DRMM patients (MFI 168,195 versus 477,000, p=0.0006).

**Table 2.** Distribution of immune regulatory receptors on NK cell subsets in myeloma patients

	*NDMM*	*DRMM*		*NDMM*	*DRMM*	
	Median % (range)	Median % (range)	Significant (p value)	Median MFI (range)	Median MFI (range)	Significant (p value)
**All NK cells**						
CD16	95.1	64.7	Yes (<0.0001)	466,000	126,851	Yes (0.0001)
DNAM-1	93.7	87.4	Yes (0.0223)	35,183	26,634	Yes (0.001)
PD-1	1.23	1.59	No (0.0558)	6,074	5,080	No (0.2372)
TIGIT	21.8	46.6	Yes (0.0046)	14,733	16,137	No (0.2722)
**CD56^bright^ NK cells**						
CD16						
DNAM-1	94.4	97.4	No (0.0880)	34,980	39,222	No (0.2496)
PD-1						
TIGIT	17.3	17.6	No (0.6206)	77	45	No (0.2166)
**CD56^dim^ NK cells**						
CD16	97.2	77.2	Yes (<0.0001)	477,000	168,195	Yes (0.0006)
DNAM-1	93.9	83.5	Yes (0.0021)	35,115	23,016	Yes (<0.0001)
PD-1	1.19	2.23	No (0.3003)	6,324	5,307	No (0.4737)
TIGIT	24.1	55.6	Yes (0.0006)	15,044	17,289	No (0.1227)

Flow cytometric analyses of immune regulatory receptors are presented as median percentage of cells expressing the respective molecules and median MFI. Statistical significance was tested using Mann Whitney U test for all datasets. NK=natural killer; NDMM=newly diagnosed multiple myeloma patients; DRMM=daratumumab refractory multiple myeloma patients, MFI=median fluorescence intensity.

### Expression of checkpoint molecules by NK cell subsets

In order to reveal divergent expression patterns of the receptors DNAM-1, PD-1, and TIGIT on NK cells, and the CD56bright and CD56^dim^ subsets, a comparison between the two groups of MM patients was performed. Both the percentage of the positive subsets and the receptor expression level as determined by MFI were taken into consideration.

The expression of PD-1 on NK cells was 1-2% in both groups (Table 2). We found no difference in its expression level, either when looking at the total NK cell compartment or in the CD56^dim^ subset. The expression of PD-1 on CD56^bright^ NK cells was below the limit of quantification.

DNAM-1 was expressed on more NK cells and at a higher level in NDMM compared to DRMM (median 93.7% versus 87.4%, *p*=0.022;MFI 35,183 versus 26,634, *p*=0.0015).This was even more pronounced in the CD56^dim^ subset (median 93.9% versus 83.5%, *p*=0.003; MFI: 35,115 versus 23,016, *p*=0.0001) (Table 2 and Fig. 3).

While the level of TIGIT expression on NK cells from NDMM and DRMM were comparable (MFI16,137 versus 14,733, p=0.272), the percentage of NK cells expressing this marker was significantly higher in the DRMM group (median 46.6% versus 21.8%, *p*=0.005) (Table 2 and Fig. 3). Again, this difference was more pronounced in the CD56^dim^ subset (median 55.6% versus 24.1%, *p*=0.001, MFI: 17,289 versus 15,044, *p*=0.123).

## Discussion

NK cells have a critical role in ADCC, which is one of the identified mechanisms of action of DARA.^7^ The immune regulatory receptors DNAM-1, PD-1, and TIGIT are expressed by NK cells, and interact with their ligands upon NK cell receptor ligation, resulting in negative or positive regulation of NK cell activity. Here, we examined the presence of these molecules on NK cell subsets from NDMM and DRMM, in order to identify changes that could relate to resistance to treatment with DARA.

Previous studies have shown that the number of circulating NK cells decreases in patients treated with DARA, and that this decrease is more pronounced for the CD56^dim^ subset.^13–15^ In line with this, we found a significant difference in reduction of the percentage of NK cells out of the total number of lymphocytes, from 21.2% in the NDMM group to 5.2% in the DRMM group. We also confirmed that the decrease was even more pronounced for the CD56^dim^ subset, which comprised a smaller fraction of the NK cells in the DRMM compared to the NDMM group. Furthermore, in the DRMM group a higher percentage of these CD56^dim^ NK cells lacked the CD16 receptor, which is necessary for ADCC. Whether or not this depletion of NK cells impairs the effects of DARA is debatable. Several studies have found that the remaining NK cells in patients treated with DARA are still able to induce some level of ADCC *in vitro*.^15,16^ In fact, Casneuf et al. found that *in vitro* ADCC was reduced after DARA treatment, but there was no difference in *in vitro* ADCC performed by PBMCs isolated from patients who responded to DARA (partial response or better) and patients with no response to DARA.^15^ Part of the explanation for this finding could be that the observed cytotoxicity *in vitro* was mediated by other cell types and mechanisms of action. However, it is also possible that the remaining NK cells retain sufficient functionality, suggesting that the reduction in frequency and receptor expression level may not have major immunological consequences and, thus, although it is likely implicated, it may not be the key factor driving DARA resistance.

In agreement with prior observations, this study showed no difference in the expression of PD-1 on the NK cell compartment from NDMM and DRMM.^21^ We have previously examined the expression of PD-1 on T-cells from the same patient groups and found no difference.^22^ Thus, the PD-1—PD-L1 axis may not be the key signaling pathway driving the development of resistance to DARA.

Contrary to the results on PD-1, the other inhibitory checkpoint receptor we examined, TIGIT, was overexpressed on NK cells from DRMM patients. These NK cells also had lower expression of the stimulatory checkpoint receptor DNAM-1 and the activation marker CD16. These differences were even more pronounced within the CD56^dim^ subset. A prior study has found a similar result with NK cells from DARA-refractory patients expressing high levels of the inhibitory checkpoint receptor TIM-3 and low levels of CD16, compared to patients with partial response or better.^21^ Collectively, these data point to phenotypic alterations of NK cells from DARA-refractory patients, suggesting a possible role for NK cells in reflecting, or perhaps even contributing, to treatment resistance.

In this study, only PB, but not BM, samples were collected. It is important to remember that PB is only a reflection of the true tumor microenvironment in the BM. With that in mind, Casneuf et al. showed that the changes in NK cells were alike in PB and BM, and in a prior study, we have examined checkpoint molecules on T cells and found no differences between PB and BM .^15,22^ Both studies implicate that NK cells from PB samples give a reasonable impression of the BM condition.

This is a hypothesis-generating study with some clear limitations due to the small sample-sizes and the lack of longitudinal data. A key limitation to this study is the comparator group consisting of newly diagnosed MM patients. This group has never been exposed to any MM treatment that might have affected their NK cell phenotype. For future studies, it will be relevant to compare the data from the DRMM group to DARA-treated responders or heavily treated patients who have not yet been exposed to DARA. The inclusion of the latter group would inform whether the exhausted-associated phenotype is linked to prior therapy burden or to DARA specifically. Furthermore, the NDMM group was older than the DRMM in our series. The age difference could also affect the NK phenotype.

Everything considered, this is an exploratory study which provides supportive but not definitive evidence for NK cell exhaustion in DARA resistance.

### Authors’ Contribution

Conceptualization: Katrine Fladeland Iversen (Equal), Line Nederby (Equal), Torben Plesner (Equal). Formal Analysis: Katrine Fladeland Iversen (Equal), Line Nederby (Equal). Funding acquisition: Katrine Fladeland Iversen (Equal), Torben Plesner (Equal). Visualization: Katrine Fladeland Iversen (Equal), Line Nederby (Equal). Writing – original draft: Katrine Fladeland Iversen (Lead). Methodology: Line Nederby (Lead). Writing – review & editing: Line Nederby (Equal), Thomas Lund (Equal), Trine Andreasen Leth (Equal), Torben Plesner (Equal). Supervision: Torben Plesner (Lead).

### Competition of Interest – COPE

All authors declare not having any competing interests.

### Ethical Conduct Approval – Helsinki – IACUC

The study was conducted according to the guidelines of the Declaration of Helsinki, and approved by the Regional Committees on Health Research Ethics for Southern Denmark (S-201702012, 02Feb2018).

### Informed Consent Statement

All authors and institutions have confirmed this manuscript for publication.

## Data Availability

All are available upon reasonable request.
